# Sensitivity of the Transport of Plastic Nanoparticles to Typical Phosphates Associated with Ionic Strength and Solution pH

**DOI:** 10.3390/ijms23179860

**Published:** 2022-08-30

**Authors:** Xingyu Liu, Yan Liang, Yongtao Peng, Tingting Meng, Liling Xu, Pengcheng Dong

**Affiliations:** School of Resources, Environment and Materials, Guangxi University, Nanning 530004, China

**Keywords:** plastic nanoparticles, phosphates, solution chemistry, retention, release

## Abstract

The influence of phosphates on the transport of plastic particles in porous media is environmentally relevant due to their ubiquitous coexistence in the subsurface environment. This study investigated the transport of plastic nanoparticles (PNPs) via column experiments, paired with Derjaguin–Landau–Verwey–Overbeek calculations and numerical simulations. The trends of PNP transport vary with increasing concentrations of NaH_2_PO_4_ and Na_2_HPO_4_ due to the coupled effects of increased electrostatic repulsion, the competition for retention sites, and the compression of the double layer. Higher pH tends to increase PNP transport due to the enhanced deprotonation of surfaces. The release of retained PNPs under reduced IS and increased pH is limited because most of the PNPs were irreversibly captured in deep primary minima. The presence of physicochemical heterogeneities on solid surfaces can reduce PNP transport and increase the sensitivity of the transport to IS. Furthermore, variations in the hydrogen bonding when the two phosphates act as proton donors will result in different influences on PNP transport at the same IS. This study highlights the sensitivity of PNP transport to phosphates associated with the solution chemistries (e.g., IS and pH) and is helpful for better understanding the fate of PNPs and other colloidal contaminants in the subsurface environment.

## 1. Introduction

Plastic nanoparticles (PNPs) are commonly defined as plastic debris smaller than 1 μm in diameter across its widest dimension and distinct from the larger microplastics (1–5000 μm) and macroplastics (larger than 5000 μm) [1]. It is reported that more than 300 million tons of plastics are manufactured each year [2,3]. The sources of PNPs in the environment may come from various materials and processes related to our daily life such as synthetic fibers [4], personal care products [5,6,7], washing [8], and packaging [9,10]. PNPs in the environment can be primary materials or degradation products of large plastic wastes as secondary production [11]. In addition, the wide application of agricultural mulch in farms or greenhouses, irrigation with waters containing plastics, and the use of sewage sludge all potentially bring a significant amount of plastics into soils [12,13,14,15,16]. Previous studies reported that the average amount of plastics in soils in southwestern China was as high as 18,760 particles per kilogram [17]. It is estimated that the annual total amount of plastics in European and North American farmlands can reach 44,000–430,000 tons per year [13].

The toxicity of PNPs to the ecosystem has been studied [18,19]. The presence of PNPs may influence the physical (e.g., hydraulic and pore distribution), chemical (e.g., contaminant adsorption), and biological properties (e.g., microbial communities) of soils [3,20,21]. Previous studies confirm that PNPs in the soil can affect the transportation, reproduction, and metabolism of soil biota [22,23]. In addition, microorganisms can act as carriers that transfer PNPs from soil to plants and eventually to other organisms through food chains [24]. Studies indicated that PNPs can pass important biological barriers (e.g., the intestinal barrier, blood–air barrier, blood–brain barrier, and placental barrier) and potentially produce adverse effects on human beings [3,25]. Furthermore, PNPs can adsorb other pollutants (organic and inorganic) and facilitate their mobility in the aqueous environment or soils thus increase the risk of coupled contaminations in the environment and groundwater [26]. PNPs show colloidal properties and are less affected by gravity due to their light weight and long-term durability [27]. The transport of PNPs in the subsurface environment is expected to be highly affected by a wide range of processes, including sedimentation, aggregation, re-suspension, and entrapment [28,29]. These processes are significantly influenced by the properties of PNPs (i.e., particle size and surface properties), solution chemistries (i.e., ionic strength (IS), cation type, and pH), porous media (i.e., grain size and surface heterogeneity), flow condition, and coexisting pollutants [30,31,32,33]. However, the transport behaviors of PNPs and the mechanisms involved are still far from being fully understood. 

Phosphates are ubiquitous in agricultural drainage and municipal wastewater [34,35] and may reach high levels in surface water and groundwater, e.g., ranging from 0.0035 to 0.1 mM after a long-term accumulation [36,37,38,39]. Furthermore, phosphate is also abundant in soils due to the wide application of phosphate fertilizers and sewage sludge on farms [38]. The presence of phosphates in the environment inevitably alters the composition of solution chemistry and the properties of the natural collector surface (i.e., soil grain surface), thus influencing colloid transport [40]. It has been demonstrated that phosphates can facilitate the transport of graphene oxide, nTiO_2_, and ZnO-NPs by increased electrostatic repulsion and the competition for retention sites between colloids and phosphates [38,41,42]. However, the influence of phosphates on the interaction of plastic particles and collector surfaces is still rarely studied and poorly understood. In addition, due to the high burdens of both phosphates and PNPs in soils, the influence of phosphates can be a critical issue in the fate of PNPs in the subsurface environment. To the best of our knowledge, the relevant information has not been reported.

Therefore, the objective of this study was to explore the potential coupled effects of typical phosphates (NaH_2_PO_4_ and Na_2_HPO_4_) associated with solution pH, ionic strength, and the presence of NaCl on the transport behaviors of PNPs using column experiments. Batch adsorption experiments, interaction energy calculations based on the classic Derjaguin–Landau–Verwey–Overbeek (DLVO) theory [43,44], and numerical simulations were also performed to better deduce the mechanisms of PNP transport. Findings in this study are helpful for better understanding the fate of PNPs and other colloidal contaminants in the subsurface environment.

## 2. Results and Discussion

### 2.1. Characterization of PNPs and Porous Media

Tables S1–S3 summarize the zeta potentials of PNPs, porous media, and hydrodynamic diameters (*d_p_*) of PNPs under all experimental conditions. In general, the *d_p_* values of PNPs in NaH_2_PO_4_ or Na_2_HPO_4_ were similar. In the absence of NaCl, the *d_p_* of PNPs did not increase much when phosphates increased from 0 to 1 mM under pH 7. Under the same phosphate concentration and pH, the *d_p_* of PNPs in the presence of NaCl was slightly larger than that without NaCl; e.g., when pH was 7, *d_p_* ranged from 124 to 150 nm and from 131 to 164 nm in NaH_2_PO_4_ and NaH_2_PO_4_–NaCl systems, respectively (Table S1). The *d_p_* values were in a larger range between 131 and 200 nm in the Na_2_HPO_4_–NaCl system under pH 7 (Table S2). The *d_p_* was also slightly larger under pH 7 compared with pH 10 under the same electrolyte. However, in 0.25 mM NaH_2_PO_4_ with 1 mM NaCl, *d_p_* was stable (128–135 nm) when the pH increased from 5 to 10. Generally, the *d_p_* was in the order of pH 10 < pH 7 with phosphate alone < pH 7 with both phosphate and NaCl. These results indicate that the charge screening under a higher IS and deprotonation of the surface hydroxyl groups under a higher pH [42] play important roles in the charge of the PNP surface.

The trends of PNP zeta potentials in two phosphates showed only minor differences. Although slight fluctuation occurred, the zeta potentials of PNPs were less negative at a higher IS as phosphate concentrations increased (less than 1 mM) with or without 1 mM NaCl and sometimes tended to become more negative under 1 mM phosphates (Tables S1 and S2). Conversely, the zeta potentials of porous media became more negative with increasing IS. This may arise from the adsorption of phosphates that creates charge density due to the deprotonation of the phosphate [45]. Figures S3 and S4 demonstrate the adsorption capacities of sand and PNPs for phosphate increased with the increase in phosphate concentrations, while the adsorption on PNPs is higher than that on sand under comparable phosphate concentrations as in column experiments (Figure S4). The adsorption behaviors can be attributed to the irreversible chemical absorption (hydrogen bonding) on quartz sand [45,46] and the reversible physical absorption on PNPs. The trends of more negative charge with increasing IS in a low-level range were also reported in previous studies for PNPs [47] and porous media [48]. Table S3 shows that the zeta potentials of PNPs and porous media under IS = 1 mM with the mixture of 0.3 mM Na_2_HPO_4_ and 0.1 mM NaCl were more negative than those of 1 mM NaCl. In addition, the zeta potentials of both PNPs and sand were more negative as pH increased due to the deprotonation of the surfaces. Values of *d_p_* and zeta potentials provided in Tables S1–S3 were used to determine the interaction energy of PNPs–sand based on DLVO theory. Figures S5–S8 show the depths of the primary minima (*Φ*_1min_), the secondary minima (*Φ*_2min_), the energy barrier height (*Φ_max_*), and the energy barrier to detachment (Δ*Φ_d_*). According to DLVO theory, larger *Φ_max_* values indicate stronger repulsions between two surfaces. Figures S5 and S6 indicate that the *Φ_max_* decreases and the depths of *Φ*_2min_ are deeper when phosphate concentrations increase. In the presence of phosphate with or without NaCl, the *Φ_max_* values of Na_2_HPO_4_ were higher than those of NaH_2_PO_4_. The *Φ_max_* tends to be larger under a higher pH (Figure S7) or under Na_2_HPO_4_ in the mixture of NaCl and phosphates at an IS of 1 mM (Figure S8). The shallow *Φ*_2min_ values indicate a low potential of retention in a secondary minimum. The *Φ*_1min_ is deeper and Δ*Φ_d_* is increased as phosphate concentration or IS increases or under lower pH, implying the potential of irreversible retention (Table S4). 

### 2.2. Transport of PNPs in the Presence of Phosphate

**Figure 1:**
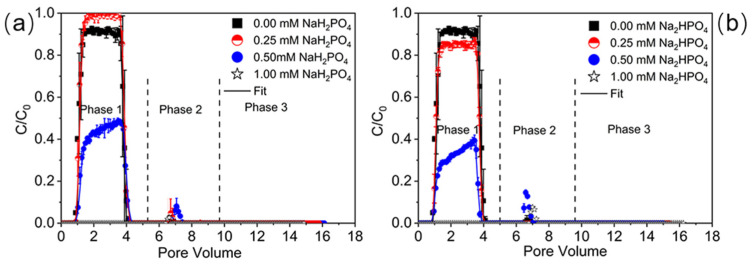
Breakthrough curves of PNPs at various NaH_2_PO_4_ (0–1 mM) (**a**) or Na_2_HPO_4_ (**b**) concentrations in the absence of NaCl under pH 7. The release of PNPs was initiated by eluting with ultrapure water under pH 7 (phase 2) and pH 10 (phase 3). Replicate experiments were performed under all experimental conditions.

Figure 1 presents the breakthrough curves (BTCs) and releases curves (RCs) of PNPs when concentrations of two phosphates equal 0.00, 0.25, 0.50, and 1.00 mM under pH 7. Table 1 shows the mass recoveries of PNPs from phases 1–3 in the column effluent. As the NaH_2_PO_4_ concentration increased from 0 to 1 mM, PNPs collected in the column effluent in phase 1 (*M*_eff_) increased from 91% to 98%, and then decreased to 43% and 0 (under detection limit). Figure 1b presents the transport of PNPs in the presence of Na_2_HPO_4_. Different from NaH_2_PO_4_, PNP transport monotonically decreased from 91% to 82%, 32.0%, and 0 as the Na_2_HPO_4_ concentration increased from 0 to 1 mM. In general, at the same concentrations of the two phosphates, PNP mobility under Na_2_HPO_4_ (Figure 1b) was much weaker than under NaH_2_PO_4_ (Figure 1a), mainly due to the higher IS of Na_2_HPO_4_ that resulted in a more pronounced compression of the electrical double layer and a reduction in repulsive force. However, when phosphate concentration was 1 mM, no breakthrough of PNPs occurred under both phosphates. Fitted values of *k*_1_ and *S_max_/C*_o_ also indicate the non-monotonic and monotonic (increased) trends in the mass transfer rates and retention capacities as NaH_2_PO_4_ and Na_2_HPO_4_ increase, respectively. However, the calculated energy barriers (*Φ_max_*) show fluctuations as phosphates increase. This deviation is attributed to the similar fluctuations of the zeta potentials for PNPs and sand that also display different trends (Tables S1 and S2). The dispersive distribution of PNPs on the sand surface shown in SEM images (Figure S9) is in agreement with *d*_p_ measurements that indicate insignificant differences in the particle size under the used IS (Tables S1 and S2). This observation indicates that aggregation is insignificant for enhanced retention and that potential physical straining under a higher IS can be excluded within the tested range of phosphate concentrations. Note that column experiments exhibit good reproducibility (Figure 1) with small standard deviations (less than 5%) for the mass recoveries in the effluent.

Previous studies demonstrated the enhanced transport of colloids (e.g., graphene oxide, TiO_2_, and ZnO NPs) in the presence of abundant NaH_2_PO_4_ or K_2_HPO_4_ under a broad concentration range (e.g., 0.1–10 mM) [38,41,42,49]. In contrast, it was also evident that the transport of TiO_2_ NPs would be reduced by increasing NaH_2_PO_4_ when the phosphate was higher than 1 mM because of the compressed electrical double layer [50]. Different from these trends, as described above, our findings suggest non-monotonic or monotonic decreased trends of PNP transport as phosphate concentrations increase in a narrow range of 0–1 mM and exhibit significant sensitivity. In particular, except for the condition of 0.25 mM NaH_2_PO_4_, the presence of phosphates under selected experimental parameters tended to inhibit PNP transport. The zeta potentials of porous media became slightly more negative as phosphate concentration increased at low levels (Tables S1 and S2). The enhanced transport that occurred in the presence of 0.25 mM NaH_2_PO_4_ can be explained by the competition for retention sites and the increased electrostatic repulsion attributed to the absorption of the phosphates, which can function as proton donors for hydrogen bonding on the collector surface [38,41,42]. However, the charge screening and the compression of the electrical double layer under a higher IS became more significant, as demonstrated by the less negatively charged PNPs and the greater retention with increasing Na_2_HPO_4_ and 1 mM NaH_2_PO_4_. Results from adsorption experiments show that the adsorption capacity of phosphate by quartz sand (Figure S3) is only up to 0.01 mg g^−1^. In comparison, higher adsorption of phosphate onto PNPs (Figure S4) reaches a value of 137 mg g^−1^ due to the larger specific surface area of PNPs than sand. RCs in Figure 1 show a minimal release of PNPs. The greater release occurred when a larger number of PNPs were retained in the previous phase (phase 1). In particular, a small portion of PNPs, which were retained under 1 mM phosphates in phase 1, can be released with the elution of ultrapure water in phase 2, whereas no release was observed in both phases when phosphates in phase 1 were less than 0.5 mM. These observations and DLVO calculations (Table S4 and Figure S5) demonstrate that most PNPs were captured in irreversible retention sites and mainly retained in deep primary minima. The negligible release is consistent with the strong energy barriers to detachment (Δ*Φ_d_*). Therefore, although the presence of NaH_2_PO_4_ may facilitate PNP transport under specific concentrations (e.g., 0.25 mM), the compression of the electrical double layer and reduced repulsive force play essential roles, leading to pronounced irreversible retention when IS reaches a threshold. Additionally, certain degrees of micro- and nanoscale surface roughness are demonstrated by SEM images in Figure S9. The presence of phosphates can also increase the surface charge/chemical heterogeneity on the solid–water interfaces. It has been well demonstrated that the surface heterogeneities of colloids and collectors tend to reduce and/or eliminate energy barriers at electrostatically unfavorable locations, thus inhibiting colloid retention [51,52,53,54]. Consequently, these surface physicochemical heterogeneities can contribute to the significant sensitivity of PNP transport to phosphate concentration and the deviations of DLVO predictions from BTCs. Therefore, PNP retention in the presence of phosphate was mainly influenced by the coupled effects of increased electrostatic repulsion, competition for retention sites, electrical double layer compression, and increased chemical heterogeneity on the interacting surfaces, depending on the types of phosphates and their concentrations.

### 2.3. Transport of PNPs in the Presence of Phosphate Mixed with NaCl

**Figure 2:**
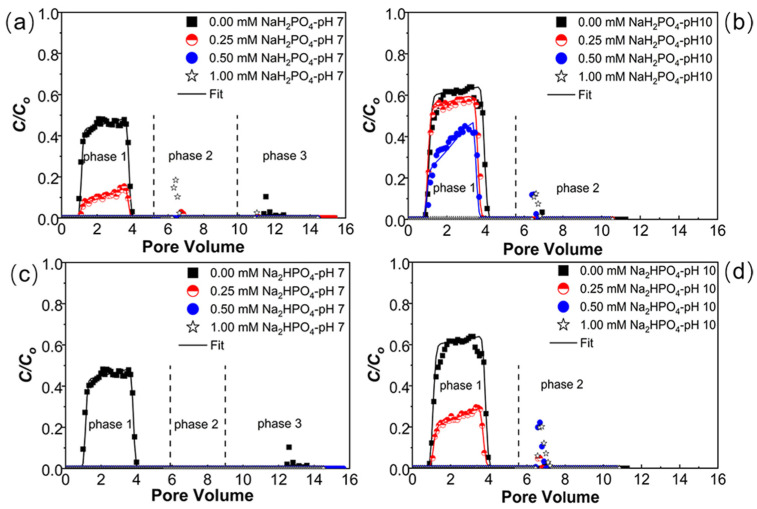
Breakthrough curves of PNPs with NaH_2_PO_4_ (0–1 mM) under pH = 7 (**a**) or pH = 10 (**b**); breakthrough curves of PNPs with Na_2_HPO_4_ (0–1 mM) at pH = 7 (**c**) or pH = 10 (**d**). All the experiments were carried out in the presence of 1 mM NaCl. The release of PNPs was initiated by eluting with ultrapure water under pH 7 (phase 2) and pH 10 (phase 3), respectively. Only the elution of water at pH 10 (phase 2) was performed when the PNPs were retained under pH 10 in phase 1.

To further investigate the influence of phosphate and IS, transport experiments were performed under 0–1 mM NaH_2_PO_4_ (Figure 2a,b) or Na_2_HPO_4_ (Figure 2c,d) at pH = 7 or 10 in the presence of 1 mM NaCl (phase 1). The release of retained PNPs was also carried out with the elution of ultrapure water under pH 7 (phase 2) and pH 10 (phase 3). Note that only the elution with water at pH 10 (phase 2) was performed in release experiments when the PNPs were retained under pH 10 in phase 1. Under pH 7 and 1 mM NaCl without phosphate, the recovery of PNPs in column effluent was 45%, whereas, under the phosphate and NaCl mixture, the PNP transport was significantly reduced. For example, at 0.25 mM NaH_2_PO_4_ with 1 mM NaCl, the *M*_eff_ decreased to 10%, and complete retention occurred when NaH_2_PO_4_ was 0.5 mM or with a higher concentration (Table 1 and Figure 2a). The retention of PNPs was more sensitive to the mixture of Na_2_HPO_4_ and NaCl under pH 7 (Figure 2c). To be specific, the *M*_eff_ was dramatically decreased to 0 when Na_2_HPO_4_ was 0.25 mM or higher. The more pronounced PNP retention with the presence of Na_2_HPO_4_ may also be attributed to the higher IS, even though the concentrations of the two phosphates were the same (Figure 2a,c). The greater PNP retention under a higher IS further demonstrated the importance of electrical double layer compression. The model was able to describe the BTCs well, and the values of *k*_1_ and *S_max_/C*_o_ increased as the IS increased (Table S5), suggesting an increasing tendency for PNP retention. These results can also be explained by the DLVO interaction energy calculations. The repulsive energy barrier (*Φ_max_*) declined as phosphate concentration increased, indicating that more PNPs overcame the energy barrier and were retained in the primary minimum in the phosphate–NaCl mixture. The asymmetric shapes of BTCs at higher phosphate concentrations reflect a more pronounced blocking effect due to the gradual filling of retention sites. These influences of the two phosphates will be further discussed below in Section 3.4.

Figure 2 indicates that the transport of PNPs under pH 10 is considerably higher than that of PNPs at pH 7. Specifically, the *M*_eff_ values of PNPs dropped from 56% without phosphate to 35% and 24% with 0.5 mM NaH_2_PO_4_ and Na_2_HPO_4_ under pH 10, respectively. However, the *M*_eff_ value of PNPs declined from 45% to under the detection limit at 0.5 mM phosphate (NaH_2_PO_4_ and Na_2_HPO_4_) under pH 7. Generally, the zeta potentials of PNPs and porous media (Tables S1 and S2) are more negative at pH 10 (compared with 7) due to deprotonation of the surface, corresponding to stronger energy barriers and shallower primary minima in DLVO calculations (Table S4 and Figure S6). Note that the increase in phosphate concentration also enhances the PNP retention in the alkaline condition, suggesting that PNP transport is sensitive to the presence of phosphate. Thus, the electrical double layer compression is still one of the main factors that influence PNP transport. The influence of solution pH on PNP transport is further discussed in Section 3.4. Similar to the retention in the presence of phosphate without NaCl, the reversible retention in phosphate and NaCl mixtures also accounted for negligible fractions (Figure 2). Only small fractions of PNPs were released in phase 2, when they were retained in the mixture of 1 mM NaH_2_PO_4_ and 1 mM NaCl under pH 7 (phase 1), and in phase 3, when the PNPs were retained without the presence of phosphates (phase 2). At pH 10 (phase 1), the release also occurred when the PNPs were previously retained under 0.25 and 0.5 mM phosphates with 1 mM NaCl. This minor release of retained PNPs further indicated that the interactions of PNPs and quartz sand were strong enough to overcome the forces of diffusion arising from hydrodynamic shear and/or random kinetic energy fluctuations [55].

### 2.4. Transport of PNPs under Various Solution pH Levels and Electrolyte Compositions 

**Figure 3:**
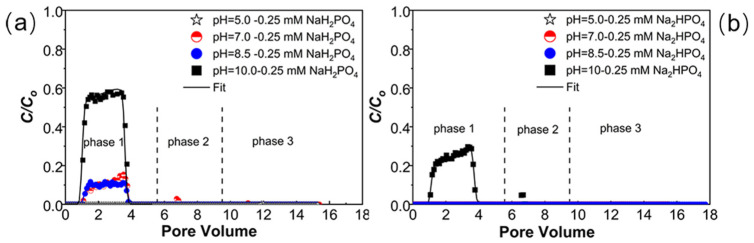
Breakthrough curves of PNPs at NaH_2_PO_4_ (0.25 mM) under pH = 5–10 (**a**); breakthrough curves of PNPs at Na_2_HPO_4_ (0.25 mM) under pH = 5–10 (**b**). All the experiments were carried out in the presence of 1 mM NaCl. The release of PNPs was initiated by eluting with ultrapure water under pH 7 (phase 2) and pH 10 (phase 3), respectively.

Figure 3 and Table 1 present experimental results of PNP transport in the presence of 0.25 mM NaH_2_PO_4_ (a) or Na_2_HPO_4_ (b) with 1 mM NaCl at various levels of solution pH (5–10). As shown in Figure 3, the retention of PNPs decreased as the solution pH increased from 5 to 10, under both conditions of NaH_2_PO_4_ and Na_2_HPO_4_. In the presence of NaH_2_PO_4_–NaCl mixture, no breakthrough occurred under pH 5.0, whereas the *M*_eff_ values were around 10% under pH 7.0 and 8.5 and dramatically rose to 53% at pH 10. In addition, with the Na_2_HPO_4_–NaCl, the *M*_eff_ was under the detection limit when the pH was 8.5 or lower but increased to 24% under pH 10. The DLVO prediction certifies the trends of increasing energy barriers/repulsion as pH increases (Table S4 and Figure S7) and is consistent with *M*_eff_ values.

Previous studies evidenced various effects of solution pH on colloid transport in the presence of phosphates. For example, the transport of ZnO NPs is negligibly influenced by pH when the K_2_HPO_4_ is abundant in the solution [38]. However, the presence of NaH_2_PO_4_ can slightly reduce the transport of TiO_2_ NPs under a higher pH due to less adsorption of phosphate [49]. In contrast, enhanced transport of graphene oxide NPs occurs as pH increases because of increasing electrostatic repulsion [41]. The forms of phosphates depend on solution pH. In particular, when the solution pH is in a range of 2.2 to 7.2, H_2_PO_4_^−^ is the major fraction of phosphates, while in pH 7.2 to 12.3, HPO_4_^2−^ and PO_4_^3−^ become the main forms, and PO_4_^3−^ is more important when the pH is higher than 10 [56,57]. Therefore, the major fractions of phosphates are independent of the initial phosphate forms when the concentrations of NaH_2_PO_4_ and Na_2_HPO_4_ are the same and under the same solution pH. With the same phosphate concentrations, the stronger retention of PNPs under Na_2_HPO_4_ than under NaH_2_PO_4_ is mainly attributed to the higher IS of Na_2_HPO_4_ (higher Na^+^ concentration), which leads to more pronounced electrical double layer compression, and surface chemical heterogeneity. As pH increases to 10, excessive OH^−^ on the surfaces of PNPs and porous media enhances the negativity of the surfaces (Tables S1 and S2) due to the enhanced magnitude of deprotonation [58]. This process intensifies the repulsive interactions between the interacting surfaces [59,60]. Consequently, the increase in PNP transport with an increased pH is mainly due to the enhanced deprotonation.

**Figure 4:**
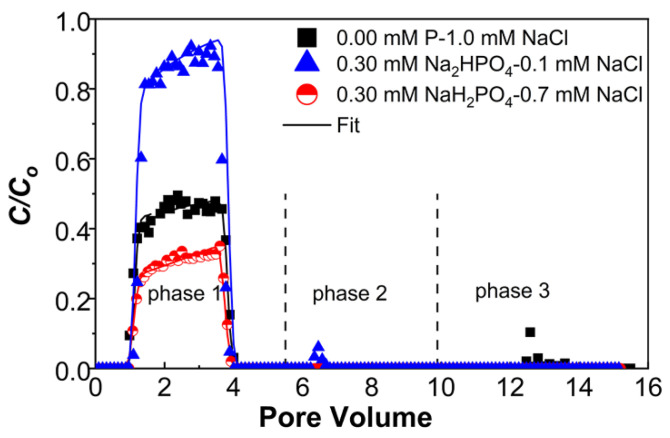
Breakthrough curves of PNPs under IS = 1 with different combinations of phosphate and NaCl at pH = 7. The release of PNPs was initiated by eluting with ultrapure water under pH 7 (phase 2) and pH 10 (phase 3), respectively.

To further deduce the combined influence of phosphates associated with IS and electrolytes on PNP transport, column experiments were conducted under the same phosphate concentration and IS under pH 7; e.g., 0.3 mM NaH_2_PO_4_ (IS = 0.3 mM) was mixed with 0.7 mM NaCl, and 0.3 mM Na_2_HPO_4_ (IS = 0.9) was mixed with 0.1 mM NaCl, to keep a constant total IS of 1 mM. Experimental results showed that *M*_eff_ accounted for 30% and 86% under 0.3 mM NaH_2_PO_4_–0.7 mM NaCl and 0.3 mM Na_2_HPO_4_–0.1 mM NaCl, respectively, compared with the 45% under 1 mM NaCl (Figure 4 and Table 1). The trend of *M*_eff_ is consistent with zeta potentials, the DLVO calculations, and fitted *S_max_/C*_o_; e.g., the calculated energy barrier between PNPs and sand is highest (Table S4) and the *S_max_/C*_o_ is smallest at 0.3 mM Na_2_HPO_4_–0.1 mM NaCl, compared to other conditions tested in Figure 4 (Table S5). The larger amount of cations (Na^+^) in 0.3 mM NaH_2_PO_4_–0.7 mM NaCl mixture could be adsorbed in the diffuse layer by electrostatic force and decreased the surface charges from inner-sphere P-adsorption [50], thus reducing the repulsive force between PNPs and sand, leading to enhanced PNP retention. Additionally, compared to NaH_2_PO_4_ under the same concentration, Na_2_HPO_4_ forms less hydrogen bonding with quartz due to the sole hydrogen bonding donors [61], and its two P-O^−^ units share more negative charge. Therefore, the interaction of Na_2_HPO_4_ and quartz will increase the electrostatic repulsion because the intermediate will carry more negative charges on oxygen [34]. Consequently, with the presence of 0.3 mM Na_2_HPO_4_–0.1 mM NaCl, the increased repulsion and the weaker charge screening/heterogeneity lead to the greatest mobility, as shown in Figure 4. In contrast, the compression of the double layer, charge screening, and the chemical/charge heterogeneity generated by the adsorption of cations will be more pronounced with an increase in Na^+^ concentration [62,63], leading to high retention of PNPs under 0.3 mM NaH_2_PO_4_–0.7 mM NaCl. These findings suggest that the association and coupled effects of electrostatic repulsion (attributed to adsorbed phosphates), the compression of the electrical double layer, and the surface chemical heterogeneity (contributed by the adsorption of cations) significantly influence the transport, depending on the phosphate concentrations, IS, and solution pH.

## 3. Materials and Methods

### 3.1. Solution Chemistry and Porous Media

Electrolyte solutions were prepared by diluting NaH_2_PO_4_, Na_2_HPO_4_, and/or NaCl in Milli-Q water, and their pH values were adjusted to 5, 7, 8.5, or 10 using HCl or NaOH. Analytically pure quartz sand (Tianjin Guangfu Fine Chemical Research Institute, Tianjing, China) was used as porous media. The sand was purified by washing in tap water, followed by soaking in HNO_3_ (65%) and H_2_O_2_ (10%) for 24 h [54]. Later, the sand was washed with water again, followed by soaking in 100 mM NaCl and ultrapure water (pH 10) with ultrasonication to remove the potential attached colloidal impurities by cation exchange and expand the electrical double layer. Finally, the quartz sand was sieved within the size range between 250 µm and 380 µm. Zeta potentials of quartz sand were measured using a ZetaSizer (Nano ZS9, Malvern Instruments, Worcestershire, UK). A scanning electron microscope (SEM, ZEISS Sigma 300, Neustadt, Germany) was used to visualize the surface morphology of quartz sand and investigate the interactions of PNPs and sand surfaces. More detailed information is provided in Supplementary Materials.

### 3.2. Plastic Nanoparticles

Polystyrene nanoparticles (purchased from Suzhou Smart Nanotechnology Co., Ltd., Suzhou, China) with regularly spherical shape and a nano size of around 50 nm were used as PNPs in this study. Polystyrene nanoparticles have been frequently employed as model colloids and representative plastics [12,64,65,66,67]. The polystyrene was confirmed by an attenuated total reflectance-Fourier transforms infrared spectroscopy (ATR-FTIR) (Nicolet iS50, Thermo Fisher Scientific, Waltham, CA, USA) (Figure S1).The initial/input concentration of PNPs in this study was set as 10 mg L^−1^ by diluting a raw suspension (1 g L^−1^) into selected electrolyte solutions and then sonicating them for 20 min in an ultrasonication bath before use. The zeta potentials and hydrodynamic diameters of PNPs were also measured using the ZetaSizer. 

### 3.3. Transport and Release Experiment

Water-saturated column experiments were performed following the processes outlined in previous studies [68]. Columns made of stainless steel with a length of 12 cm and an inner diameter of 3 cm were wet-packed with purified quartz sand. A constant velocity was set as 0.7 cm min^−1^ for all experiments by a peristaltic pump that introduced PNP suspensions and PNP-free electrolyte solutions upward into the vertical columns. The columns were firstly conditioned with background solutions (30 pore volumes) of 0–1 mM NaH_2_PO_4_ or Na_2_HPO_4_ under different pH values with or without the presence of NaCl. Later, the transport of the tracer and PNPs was investigated in each column experiment by injecting a 100 mL pulse of tracer (2–4 times the concentrations of background solution) or PNP suspension, followed by elution with 100 mL background solution. Column effluent samples (4 mL of each) were continuously collected via a fraction collector. A conductivity meter and a fluorescence spectrophotometer were used to determine the concentrations and obtain the corresponding breakthrough curves (BTCs) of the tracer and PNPs, respectively. Experimental conditions are summarized in Table 1. To test the reproducibility, some experimental conditions were repeated in column experiments.

After the completion of transport experiments named phase 1, the release of retained PNPs was conducted to examine the potential detachment from the secondary or primary minimum. The retained PNPs were rinsed with several pore volumes of ultrapure water under the same pH (phase 2) as in phase 1 and then with ultrapure water at pH 10 (phase 3). Release curves (RCs) of PNPs were also determined using a fluorescence spectrophotometer. 

### 3.4. Batch Experiments and Theory

Batch adsorption experiments were performed to investigate the adsorption of phosphates on 50 nm PNPs (100 mg L^−1^) and sand (2 × 10^5^ mg L^−1^) under different phosphate concentrations (0.25–6 mM) at pH 7. The mixtures were shaken by a water bath oscillator at 25 °C for 24 h. Before the quantification of phosphates, 40 µL CaCl_2_ (2 mol L^−1^) was added to form larger PNP aggregates to overcome the challenge in the separation of PNPs from the liquid phase [69], followed by centrifugation at 15,000 rpm (23,120× *g*) for 15 min and then filtration through a 0.22 µm membrane. The phosphates were then treated and determined by a colorimetric method at a fixed wavelength of 700 nm using a visible spectrophotometer [70] (Section S2 and Figure S2 in Supporting Information). All adsorption experiments were carried out in triplicate.

Classical DLVO theory was used to calculate the interaction energy between PNPs and quartz sand under various solution chemistries as in column experiments. The total interaction energy includes electrical double layer repulsive and van der Waals attractive forces [71,72]. The transport of PNPs was described by inverse fitting to experimental BTCs to obtain the parameters of *k*_1_ and *S_max_/C*_o_ by HYDRUS-1D computer code [73]. The *k*_1_ and *S_max_/C*_o_ represent the first-order retention coefficient and the maximum solid-phase concentration of deposited PNPs, respectively. Tracer experiments were used to determine the values of dispersivity and pore water velocity in the simulations for PNP transport. The simulation did not perform when the PNP effluent concentration was under the detection limit. Further information on the DLVO interaction energy calculations and the descriptions of numerical simulations are provided in the Supplementary Information (Sections S4 and S5).

## 4. Conclusions

PNP transport is significantly sensitive to phosphate concentrations at low levels. The transport of PNPs is non-monotonically influenced by NaH_2_PO_4_ due to the increased electrostatic repulsion, the competition for retention sites, and the compression of the double layer varying with IS. The transport is inhibited when NaH_2_PO_4_ concentration reaches a threshold. However, an increase in Na_2_HPO_4_ tends to result in a monotonic decrease in PNP transport. These observations are different from the findings that show an increase in colloid transport even at a much larger range of NaH_2_PO_4_ concentrations in previous studies. Higher pH increases PNP transport due to the deprotonation of surfaces. A minimal fraction for the release of retained PNPs under reduced IS and increased pH indicate that the PNPs are mainly captured in deep primary minima on irreversible retention sites. The presence of Na_2_HPO_4_ tends to result in greater PNP retention than NaH_2_PO_4_ under the same concentration due to the fact of higher IS and cation concentration for Na_2_HPO_4_. Additionally, hydrogen bonding from two phosphates that act as proton donors contributes to variations in the interactions of PNPs and porous media and thus influences PNP transport. These findings further demonstrate that the compression of the electrical double layer tends to be dominant over the electrostatic repulsion arising from the adsorption of phosphates on the interacting surfaces. The adsorption of phosphates can also increase chemical heterogeneity, thus reducing PNP transport and increasing the sensitivity of particle transport to IS, due to the potential reduction/elimination of the energy barrier. Classical DLVO needs an extension to include the influence of physicochemical heterogeneities for a better explanation of experimental results. 

This study highlights the sensitivity of PNP transport to phosphates associated with the solution chemistry and indicates enhanced retention of PNPs in the presence of phosphate (≥1 mM), higher IS, and low pH. The findings are helpful for better understanding the fate of PNPs and colloidal contaminants in the subsurface environment. They also imply that the presence of phosphates will influence PNP transport in engineering processes (e.g., deep bed filtration), agricultural soil, or contaminated subsurface environments. However, polystyrene spheres could not perfectly represent all PNPs in the real soil environment. Further investigations on PNPs from different sources with varying shapes and surface properties are also needed for a better understanding of the environmental fate of colloidal plastics.

## Figures and Tables

**Table 1:** Experimental parameters and the mass recoveries of PNPs under various experimental conditions.

	NaH_2_PO_4_ mM	Na_2_HPO_4_ mM	NaCl mM	pH	IS	Recovery (%)		
						*M*_eff_	*M*_2_	*M*_3_
Figure 1a	0	0	0	7	0.01	91	-	-
	0.25	0	0	7	0.25	98	-	1
	0.5	0	0	7	0.50	43	1	-
	1	0	0	7	1.00	-	-	-
Figure 1b	0	0	0	7	0.01	91	-	-
	0	0.25	0	7	0.75	82	-	-
	0	0.5	0	7	1.50	32	2	-
	0	1	0	7	3.00	-	2	-
Figure 2a	0	0	1	7	1.00	45	-	1
	0.25	0	1	7	1.25	10	-	-
	0.5	0	1	7	1.50	-	-	-
	1	0	1	7	1.75	-	2	-
Figure 2b	0	0	1	10	1.00	56	-	-
	0.25	0	1	10	1.25	53	-	-
	0.5	0	1	10	1.50	35	-	1
	1	0	1	10	2.00	-	-	1
Figure 2c	0	0	1	7	1.00	45	-	1
	0	0.25	1	7	1.75	-	-	-
	0	0.5	1	7	2.50	-	-	-
	0	1	1	7	4.00	-	-	-
Figure 2d	0	0	1	10	1.00	56	-	-
	0	0.25	1	10	1.75	24	-	-
	0	0.5	1	10	2.50	-	-	2
	0	1	1	10	4.00	-	-	3
Figure 3a	0.25	0	1	5	1.25	-	-	-
	0.25	0	1	7	1.25	10	-	-
	0.25	0	1	8.5	1.25	10	-	-
	0.25	0	1	10	1.25	53	-	-
Figure 3b	0	0.25	1	5	1.75	-	-	-
	0	0.25	1	7	1.75	-	-	-
	0	0.25	1	8.5	1.75	-	-	-
	0	0.25	1	10	1.75	24	-	-
Figure 4	0	0	1	7	1.00	45	-	1
	0.3	0	0.7	7	1.00	30	-	-
	0	0.3	0.1	7	1.00	86	1	-

“-” denotes under detection limit; *M*_eff_ is the mass percentage of PNPs recovered from effluents in the retention (phase 1). *M*_2_ and *M*_3_ are the mass percentages of PNPs recovered from release phase 2 and phase 3. Note that only one release phase (elution with ultrapure water under pH = 10) was performed when PNPs were retained under pH = 10 in phase 1.

## Data Availability

The data presented in this study are available in Supplementary Material.

## Supplementary Materials

The supporting information can be downloaded at: https://www.mdpi.com/article/10.3390/ijms23179860/s1. References [74,75,76,77,78,79] are cited in the supplementary materials.
