# Supplementary material for: Sensitivity of the Transport of Plastic Nanoparticles to Typical Phosphates Associated with Ionic Strength and Solution pH
Source: Int J Mol Sci. 2022 Aug 30;23(17):9860. doi: 10.3390/ijms23179860 (PMC9455956; doi:10.3390/ijms23179860)
Supplement: Supplementary file 1 [file ijms-23-09860-s001.zip › ijms-1838657-supplementary.pdf]

Supporting Information

Sensitivity of the Transport of Plastic Nanoparticles to Typical Phosphates Associated with Ionic Strength and Solution pH

Xingyu Liu[1], Yan Liang[1*], Yongtao Peng[1], Tingting Meng[1], Lilin Xu[1], Pengcheng Dong[1]

[1]School of Resources, Environment and Materials, Guangxi University, Nanning 530004, China

Pages: 16

Table: 5

Figures: 9

* Corresponding Author. E-mail address: liangyan@gxu.edu.cn

Introduction

Supplementary information contains a brief description and discussion of: (i) Characterization of plastic nanoparticles (PNPs) (S1); (ii) Quantification of phosphates (S2); (iii) Zeta potentials measurement; (iv) Transport model (S3); (v) Interaction energy calculations (S4); (vi) The FTIR spectrum of PNPs. (Fig. S1); (vii) The standard curve of phosphate determined by visible spectrophotometer after treatments using the colorimetric method (Fig. S2); (viii) The adsorption capacity of sand for phosphate (NaH_2PO_4) at pH 7 in the presence of 1 mM NaCl (Fig. S3); (ix) The adsorption capacity of PNP for phosphate (NaH_2PO_4) at pH 7 in the presence of 1 mM NaCl (Fig S4); (x) DLVO interaction energy profiles for PNP-Sand under different concentrations of NaH_2PO_4 (a) or Na_2HPO_4 (b) without 1 mM NaCl at pH 7 (Fig S5); (xi) DLVO interaction energy profiles for PNP-Sand under different concentrations of NaH_2PO_4 or Na_2HPO_4 with 1 mM NaCl at pH 7 (a, c) and pH 10 (b, d) (Fig S6); (xii) DLVO interaction energy profiles for PNP-Sand under 0.25 mM NaH_2PO_4 (a) or Na_2HPO_4 (b) with 1 mM NaCl at pH 5-10 (Fig S7); (xiii) DLVO interaction energy profiles for PNP-Sand under the same ionic strength (IS =1) with NaH_2PO_4 or Na_2HPO_4 and NaCl at pH 7 (Fig S8); (xiv) SEM images of retained PNPs in the presence of 1 mM NaH_2PO_4 (a) or Na_2HPO_4 (b) and 1 mM NaCl under pH 7 (Fig S9); (xv) Hydrodynamic diameter (d_p) and zeta potentials of PNPs and sand under various NaH_2PO_4 concentrations with and without the presence of NaCl under a pH range of 5-10 (Table S1); (xvi) Hydrodynamic diameter (d_p) and zeta potentials of PNPs and sand under various Na_2HPO_4 concentrations with and without the presence of NaCl under a pH range of 5-10 (Table S2); (xvii) Hydrodynamic diameter (d_p) and zeta potentials of PNPs and sand under the ionic strength of 1 mM with different electrolyte combinations under pH 7 (Table S3); (xviii) The calculated DLVO interaction energy of PNPs - Sand under various experimental conditions (Table S4); (xix)

Fitted values (k_1 and S_{max}/C_o) of PNP transport under various experimental conditions obtained via inverse fitting by HYDRUS-1D computer code (Table S5).

Section S1. Characterization of plastic nanoparticles (PNPs)

The concentration of PNPs stock suspension was 1.00 g L^{-1}. The influent concentrations of PNPs were 10 mg L^{-1} by diluting the stock suspension into corresponding solutions. PNPs are identified by attenuated total reflectance-Fourier transforms infrared spectroscopy (ATR-FTIR) in the range from 500 to 4000 cm^{-1} using a Nicolet iS50 spectrometer (Thermo Fisher Scientific), e.g., the absorption bands at 3449, 2917, and 1656 cm^{-1} reflect the O-H, C-H, and C=C bond stretching, respectively (Benzene ring) [74]. This observation is similar to the previous study [75]. The diameter and morphology of PNPs were observed by dynamic light scattering (DLS, Nano ZS9, Malvern Instruments, Worcestershire, U.K.) and scanning electron microscopy (SEM, ZEISS Sigma 300, Germany).

Section S2. Quantification of phosphates

The phosphates were then treated and determined by a colorimetric method. Briefly, the supernatant is diluted by a certain factor to fit the range of the standard curve. After that, 1 mL of 100 g/L ascorbic acids was added to the supernatant, followed by the addition of a 2 mL color agent which was a mixture of 130 g/L ammonium molybdate, 3.5 g/L antimony potassium tartrate, and 48% H_2SO_4 with a volumetric ratio of 1:1:3 to form an antimony-phospho-molybdate complex. The absorbance of the solution was measured at 700 nm using a UV/Vis spectrophotometer.

Section S3. Zeta potential measurement

The zeta potentials of PNP and sands were determined by ZetaSizer (Nano ZS9, Malvern Instruments, Worcestershire, U.K.), using the Smoluchowski equation:

$$\upsilon_{\mathrm{E}} = 4\pi\varepsilon_0\varepsilon_r \frac{\zeta}{6\pi\mu}(1+\kappa\gamma) \qquad \text{[S1]}$$

Where υ_E is mobility, ζ is the zeta potentials, ε_0 and ε_r are the relative dielectric constant and the electrical permittivity of a vacuum respectively, μ is the solution viscosity, γ is the particle radius, and κ is the Deby-Hückel parameter [76].

Section S4. Transport model

To obtain the k_1 and *Smax* which represent the first-order coefficient and the maximum solid-phase concentration of deposited PNPs, experimental BTCs were inversely fitted to the PNPs transport model by computer code [73]. Advection-dispersion equation was used to describe the transport of PNPs in the aqueous phase, which includes an exchange term to/from the aqueous and the solid phases:

$$\frac{\partial(\theta_w c)}{\partial_t} + \frac{\partial(\rho_b s)}{\partial_t} = \frac{\partial}{\partial_z}\left(\theta_w D \frac{\partial_c}{\partial_z}\right) - \frac{\partial(qc)}{\partial_z} \qquad \text{[S2]}$$

where θw *[–]* is the volumetric water content, C[$Nc\ L^{-3}$, *Nc* and *L* denote the number of PNPs and units of length, respectively] is the aqueous phase PNPs concentration, *t* is time [*T*], z [*L*] is the distance from the column inlet, ρ_b [$M\ L^{-3}$], *M* denotes units of mass] is the soil bulk density, *S* [$Nc\ M^{-1}$] is the solid phase PNPs concentration, *D* [L^2T^{-1}] is the hydrodynamic dispersion coefficient, *q* [$L\ T^{-1}$] is the Darcy water flux. In Eq. [S2], the second term on the left-hand side is used to describe

PNPs retention in the solid phase, and the first and second terms on the right-hand side account for the dispersive and advective fluxes of PNPs, respectively [68].

The solid phase mass balance equation is given in this work as:

$$\frac{\partial(\rho_b s)}{\partial_t} = \theta_w \kappa_1 \psi C \qquad [S3]$$

where k_1 [T^{-1}] is the first-order retention coefficient, ψ [-] is a dimensionless function to account for time-dependent blocking/filling of retention sites using a Langmuirian approach [77] and it is given in Eq.

$$\psi = 1 - \frac{S}{S_{max}} \qquad [S4]$$

where *Smax* [$Nc\ M^{-1}$] is the maximum solid-phase concentration of deposited PNPs. Values of the Darcy velocity (*q*), porosity (*θ*), bulk density (*ρ*), and dispersivity (*λ*) were determined from available experimental information and tracer experiments[68]. Other model parameters were determined by optimization to PNPs breakthrough curves using the Levenberg-Marquardt nonlinear least-squares fitting routine in HYDRUS-1D [73].

Section S5. Interaction energy calculations

Derjaguin–Landau–Verwey–Overbeek (DLVO) theory was used to calculate the van der Waals (V_{VDW}), electrostatic double layer (V_{EDL}), and total interaction energies (*VTOT*) which include V_{VDW} and V_{EDL} of PNPs-PNPs and PNPs-quartz sand.

$$V_{TOT} = V_{VDW} + V_{EDL} \qquad [S5]$$

The van der Waals (V_{VDW}) of PNPs-PNPs and PNPs-Sand were determined by assuming sphere–sphere geometry and sphere-plate geometry. The following equations were utilized [71,72].

$$V_{VDW(PNPs-PNPs)} = -\frac{A_{101}a_p}{12h}[1+\frac{14h}{\lambda}]^{-1} \quad [S6]$$

$$V_{VDW(PNPs-Sand)} = -\frac{A_{102}a_p}{6h}[1+\frac{14h}{\lambda}]^{-1} \quad [S7]$$

The electrostatic double layer (V_{EDL}) of PNPs-PNPs and PNPs-Sand were developed treating according to the following equations:

$$V_{EDL\ (PNPs-PNPs)} = 2\pi\varepsilon_0\varepsilon a_p\psi_p^2\, ln[\,1+exp(-\kappa h] \quad [S8]$$

$$V_{EDL(PNPs-Sand)} = \pi\varepsilon_0\varepsilon a_p\{2\psi_p\psi_c\, ln[\frac{1+exp(-\kappa \hbar)}{1-exp(-\kappa \hbar)}] + (\psi_p^2+\psi_c^2)\, ln[(1 - exp(-2\kappa \hbar)]]\} \quad [S9]$$

Where, A_{101} is Hamaker constant for the PNPs-water-PNPs system; a_p is the radius of PNPs nanoparticles (m), h is the separation distance between PNPs-PNPs or PNPs-sand (m). λ usually denotes a characteristic wavelength of 100 nm. Permittivity in vacuum (ε_0) and dielectric constant (ε) of water are 8.854 × 10−12 C/V/m and 78.5 respectively. K is the inverse Debye length (m^{-1}) and can be calculated as:

$$\kappa = \sqrt{\frac{e^2\sum n_{j0}z_j^{\ 2}}{\varepsilon_0\varepsilon_r K_B T}} \quad [S10]$$

where z_j is the ion valence, e is the electron charge (-1.60×10-19 C), n_{j0} is the number concentration of ions in the bulk solution, K_B is the Boltzmann constant (1.38×10-23 J/K), T is Kelvin temperature (298 K).

A_{102} is the Hamaker constant for the PNP-water-soil, which can be defined as follows:

$$A_{101} = (\sqrt{A_{11}} - \sqrt{A_{00}})^2 \quad [S11]$$

$$A_{102} = (\sqrt{A_{11}} - \sqrt{A_{00}})(\sqrt{A_{22}} - \sqrt{A_{00}}) \quad [S12]$$

where A_{11} is the Hamaker constant for PNPs (6.6×10^{-20} J) [78], A_{00} is the Hamaker constant for water (3.7×10^{-20} J) [78]. A_2 is the Hamaker constant for quartz sand ($A_2 = 6.5 \times 10^{-20}$J) [79].

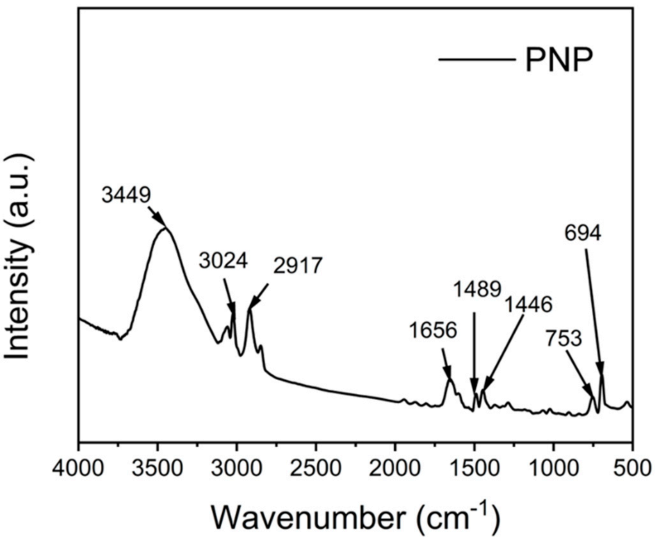

Figure S1. The FTIR spectrum of PNPs. The characteristic adsorption peaks of polystyrene are 3449, 2917, and 1656 cm^{-1}, which reflect the O-H, C-H, and C=C bond stretching, respectively.

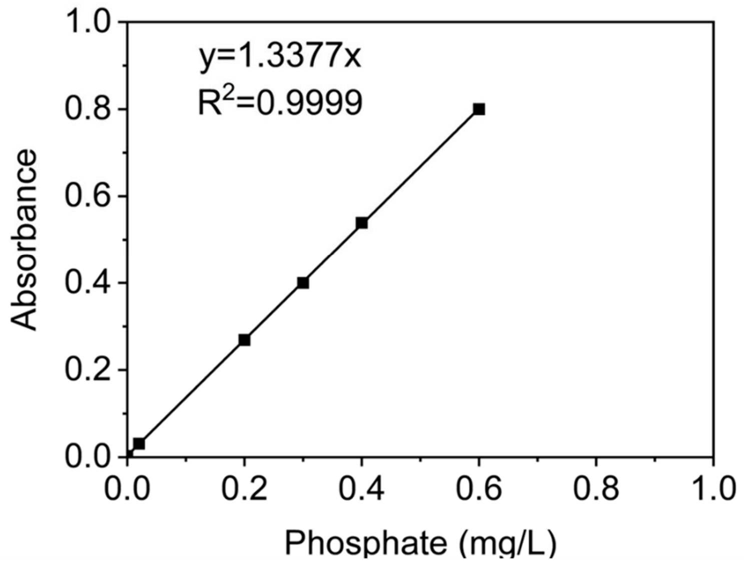

Figure S2. The standard curve of phosphate determined by visible spectrophotometer after treatments usingthe colorimetric method.

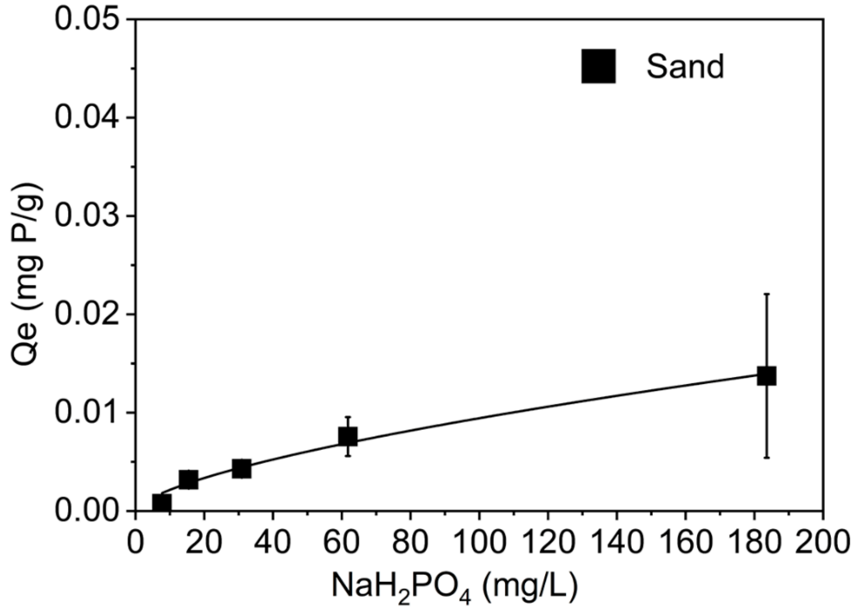

Figure S3. The adsorption capacity of sand for phosphate (NaH_2PO_4) at pH 7 in the presence of 1 mM NaCl. Q_e is the equilibrium adsorption capacity. Error bars represent standard deviations in duplicate.

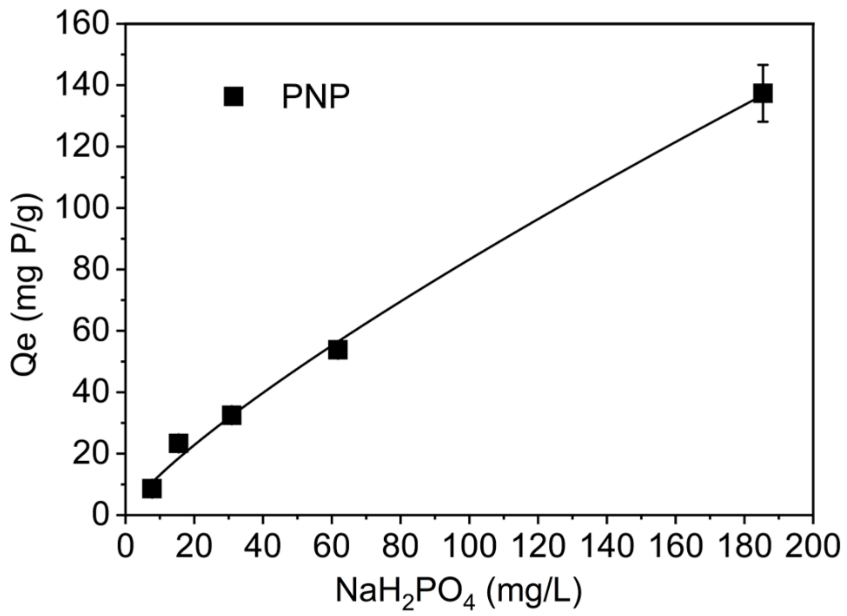

Figure S4. The adsorption capacity of PNP for phosphate (NaH_2PO_4) at pH 7 in the presence of 1 mM NaCl. Q_e is the equilibrium adsorption capacity. Error bars represent standard deviations in duplicate.

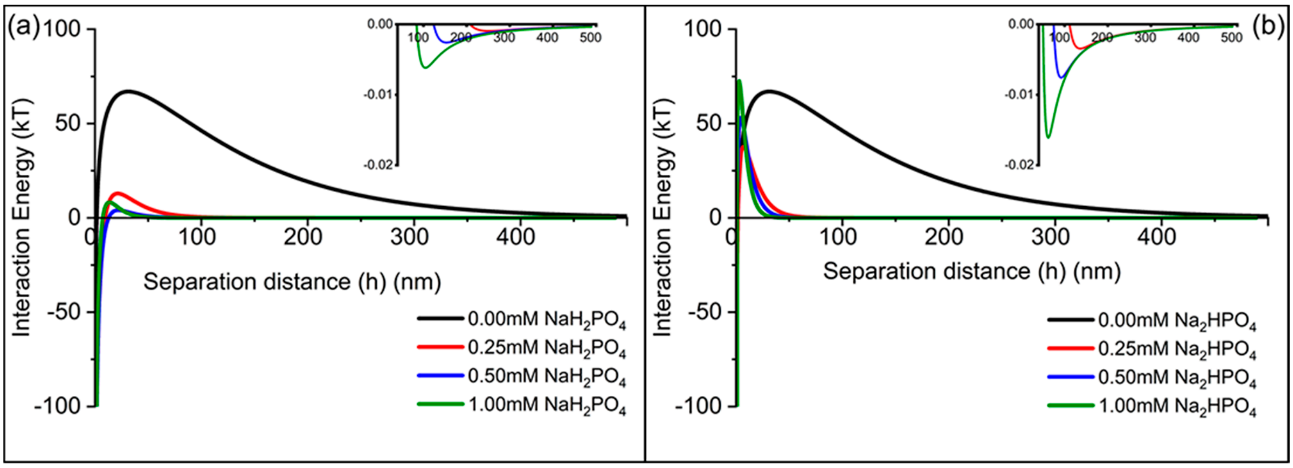

Figure S5. DLVO interaction energy profiles for PNP-Sand as a function of the separation distance under different concentrations NaH_2PO_4 (a) or Na_2HPO_4 (b) without 1 mM NaCl at pH 7.

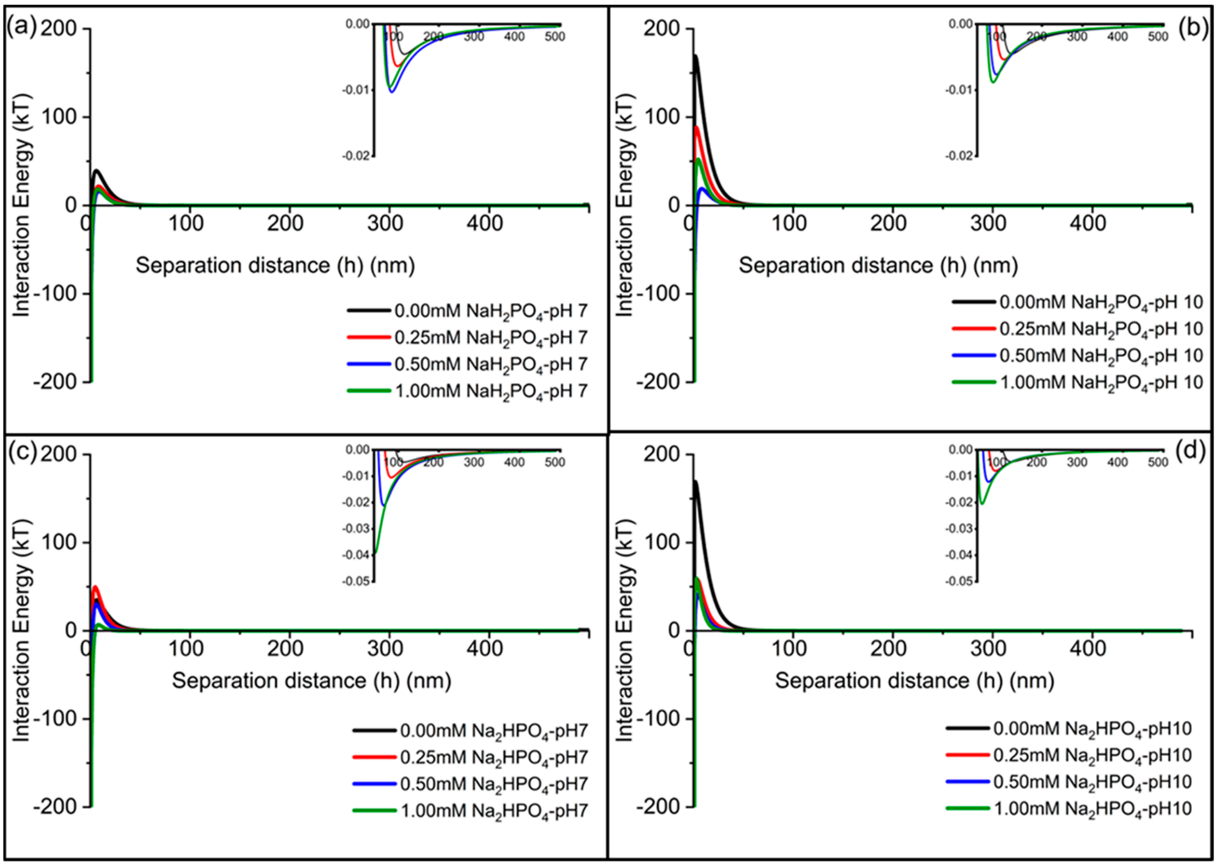

Figure S6. DLVO interaction energy profiles for PNP-Sand as a function of the separation distance under different concentrations of NaH_2PO_4 (a, b) or Na_2HPO_4 (0-1 mM) (c, d) with 1 mM NaCl at pH 7 (a, c) and pH 10 (b, d).

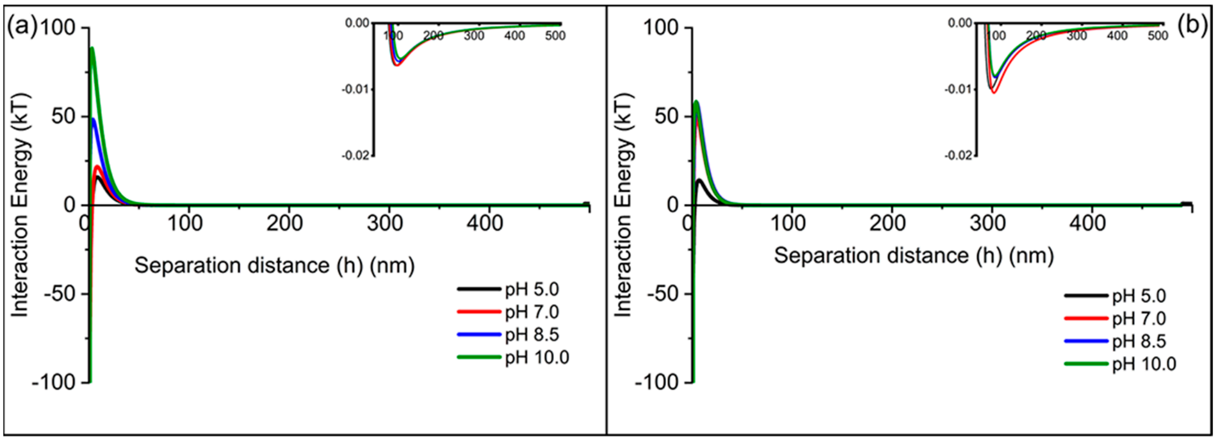

Figure S7. DLVO interaction energy profiles for PNP-Sand as a function of the separation distance under 0.25 mM NaH_2PO_4 (a) or Na_2HPO_4 (b) with 1 mM NaCl at pH 5-10.

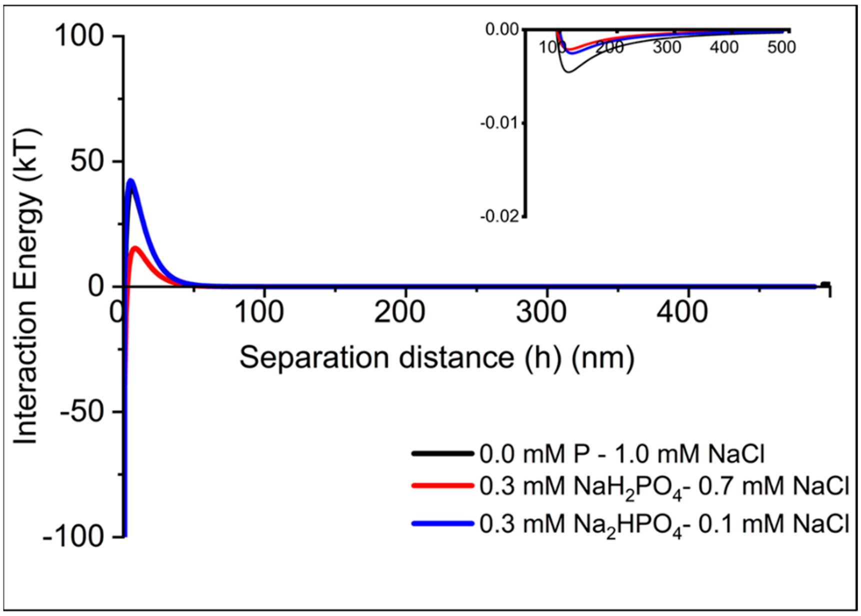

Figure S8. DLVO interaction energy profiles for PNP-Sand as a function of the separation distance under the same ionic strength (IS =1) with mixtures of phosphate (NaH_2PO_4 or Na_2HPO_4) and NaCl at pH 7.

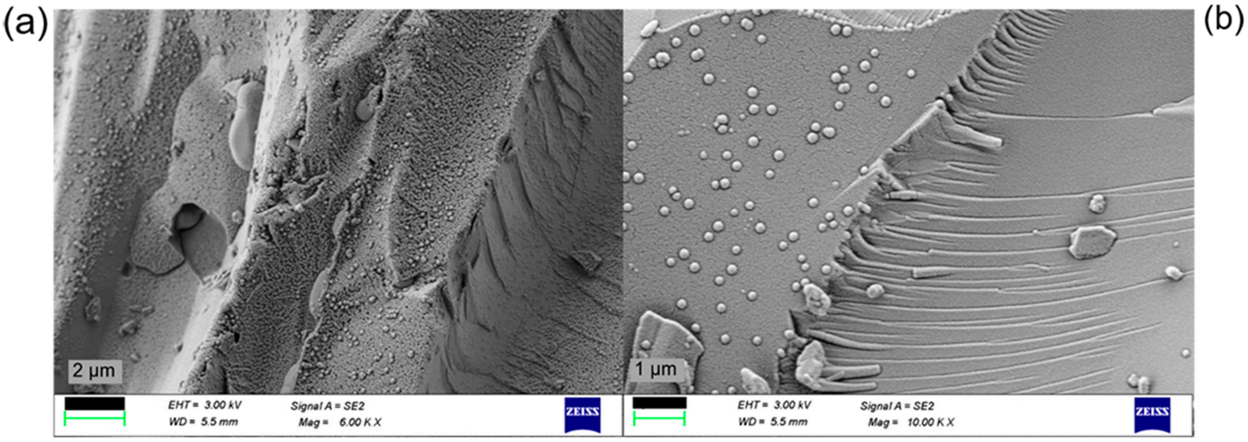

Figure S9. SEM images of retained PNPs in the presence of 1 mM NaH_2PO_4 (a) or Na_2HPO_4 (b) and 1 mM NaCl under pH 7.

Table S1. Hydrodynamic diameter (d_p) and zeta potentials of PNPs and sand under various NaH_2PO_4 concentrations with and without the presence of NaCl under a pH range of 5-10

	NaH_2PO_4 mM	NaCl mM	pH	IS	d_p(nm)	ξ_{sand} (mv)	ξ_{PNPs} (mv)
Figure 1a	0	0	7	0.01	128	-54	-23
	0.25	0	7	0.25	124	-59	-10
	0.5	0	7	0.5	131	-54	-6
	1	0	7	1	150	-59	-7
Figure 2a	0	1	7	1	131	-58	-16
	0.25	1	7	1.25	135	-62	-13
	0.5	1	7	1.5	164	-56	-10
	1	1	7	1.75	134	-62	-12
Figure 2b	0	1	10	1	117	-64	-35
	0.25	1	10	1.25	131	-58	-27
	0.5	1	10	1.5	131	-65	-12
	1	1	10	2	124	-73	-21
Figure 3a	0.25	1	5	1.25	128	-55	-11
	0.25	1	7	1.25	135	-62	-13
	0.25	1	8.5	1.25	128	-52	-20
	0.25	1	10	1.25	131	-58	-27

IS, ionic strength. d_p, the hydrodynamic diameter of PNPs. ξ_{sand} and ξ_{PNPs} are the zeta potentials of sand and PNPs, respectively.

Table S2. Hydrodynamic diameter (d_p) and zeta potentials of PNPs and sand under various Na_2HPO_4 concentrations with and without the presence of NaCl under a pH range of 5-10

	Na_2HPO_4 mM	NaCl mM	pH	IS	d_p(nm)	ξ_{sand} (mv)	ξ_{PNPs} (mv)
Figure 1b	0	0	7	0.01	128	-54	-23
	0.25	0	7	0.85	137	-57	-17
	0.5	0	7	1.5	142	-61	-120
	1	0	7	3	143	-67	-23
Figure 2c	0	1	7	1	131	-58	-16
	0.25	1	7	1.75	161	-61	-18
	0.5	1	7	2.5	200	-62	-12
	1	1	7	4	183	-64	-6
Figure 2d	0	1	10	1	117	-64	-35
	0.25	1	10	1.75	128	-64	-22
	0.5	1	10	2.5	127	-70	-18
	1	1	10	4	131	-70	-22
Figure 3b	0.25	1	5	1.75	130	-55	-11
	0.25	1	7	1.75	161	-61	-18
	0.25	1	8.5	1.75	133	-57	-21
	0.25	1	10	1.75	128	-64	-22

IS, ionic strength; d_p, the hydrodynamic diameter of PNPs; ξ_{sand} and ξ_{PNPs} are the zeta potentials of sand and PNPs, respectively.

Table S3 Hydrodynamic diameter (d_p) and zeta potentials of PNPs and sand under the ionic strength of 1 mM with different electrolyte combinations under pH 7.

	NaH_2PO_4 mM	Na_2HPO_4 mM	NaCl mM	pH	IS	d_p(nm)	ξ_{sand} (mv)	ξ_{PNPs} (mv)
Figure 4	0	0	1	7	1	131	-58	-17
	0.3	0	0.7	7	1	62	-70	-16
	0	0.3	0.1	7	1	80	-70	-23

IS, ionic strength; d_p, the hydrodynamic diameter of PNPs; ξ_{sand} and ξ_{PNPs} are the zeta potentials of sand and PNPs, respectively.

Table S4. The calculated DLVO interaction energy of PNPs- Sand under various experimental conditions.

	NaH_2PO_4 mM	Na_2HPO_4 mM	NaCl mM	pH	IS	Φ_{1min} $kT\times10^5$	Φ_{max} kT	$\triangle\Phi_d=\Phi_{max}-\Phi_{1min}$ $kT\times10^5$
Figure 1a	0	0	0	7	0.01	-1.05	67	1.05
	0.25	0	0	7	0.25	-1.02	13	1.02
	0.5	0	0	7	0.5	-1.08	4	1.08
	1	0	0	7	1	-1.24	8	1.24
Figure 1b	0	0	0	7	0.01	-1.05	67	1.05
	0	0.25	0	7	0.75	-1.13	38	1.13
	0	0.5	0	7	1.5	-1.16	53	1.16
	0	1	0	7	3	-1.17	73	1.17
Figure 2a	0	0	1	7	1	-1.08	35	1.08
	0.25	0	1	7	1.25	-1.11	22	1.11
	0.5	0	1	7	1.5	-1.35	18	1.35
	1	0	1	7	1.75	-1.11	19	1.11
Figure 2b	0	0	1	10	1	-1.24	140	1.24
	0.25	0	1	10	1.25	-1.07	88	1.07
	0.5	0	1	10	1.5	-1.08	19	1.08
	1	0	1	10	2	-1.02	52	1.02
Figure 2c	0	0	1	7	1	-1.08	35	1.08
	0	0.25	1	7	1.75	-1.33	50	1.33
	0	0.5	1	7	2.5	-1.65	30	1.65
	0	1	1	7	4	-1.51	7	1.51
Figure 2d	0	0	1	10	1	-1.24	140	1.24
	0	0.25	1	10	1.75	-1.05	58	1.05
	0	0.5	1	10	2.5	-1.05	41	1.05
	0	1	1	10	4	-1.08	60	1.08
Figure 3a	0.25	0	1	5	1.25	-1.06	16	1.06
	0.25	0	1	7	1.25	-1.11	22	1.11
	0.25	0	1	8.5	1.25	-1.05	48	1.05
	0.25	0	1	10	1.25	-1.07	88	1.07
Figure 3b	0	0.25	1	5	1.75	-1.07	14	1.07
	0	0.25	1	7	1.75	-1.33	50	1.33
	0	0.25	1	8.5	1.75	-1.09	56	1.09
	0	0.25	1	10	1.75	-1.05	58	1.05
Figure 4	0	0	1	7	1	-1.08	35	1.08
	0.3	0	0.7	7	1	-0.51	15	0.51
	0	0.3	0.1	7	1	-0.66	41	0.66

Φ_{1min}, the depths of the primary minimum; Φ_{2min}, the depths of the primary minimum; Φ_{max}, energy barrier; $\triangle\Phi_d$, the energy barrier to detachment from the primary minimum ($\triangle\Phi_d=\Phi_{max}-\Phi_{1min}$).

Table S5. Fitted values (k_1 and S_{max}/C_o) of PNP transport obtained via inverse fitting by HYDRUS-1D computer code

	NaH_2PO_4 mM	Na_2HPO_4 mM	NaCl mM	pH	IS	k_1 min^{-1}	S_{max}/C_o cm^3 g^{-1}	R^2
Figure 1a	0	0	0	7	0	0.01	0.02	0.98
	0.25	0	0	7	0.3	0.00	0.00	0.98
	0.5	0	0	7	0. 5	0.01	0.14	0.99
	1	0	0	7	1.0	-	-	-
Figure 1b	0	0	0	7	0	0.01	0.02	0.98
	0	0.25	0	7	0.75	0.02	0.07	0.99
	0	0.5	0	7	1.5	0.17	0.14	0.99
	0	1	0	7	3	-	-	-
Figure 2a	0	0	1	7	1	0.10	2.74	0.98
	0.25	0	1	7	1.25	0.33	2.25	0.98
	0.5	0	1	7	1.5	-	-	-
	1	0	1	7	2	-	-	-
Figure 2b	0	0	1	10	1	0.06	2.04	0.99
	0.25	0	1	10	1.25	0.10	0.82	0.93
	0.5	0	1	10	1.5	0.15	1.2	0.98
	1	0	1	10	1.75	-	-	-
Figure 2c	0	0	1	7	1	0.10	2.74	0.98
	0	0.25	1	7	1.75	-	-	-
	0	0.5	1	7	2.5	-	-	-
	0	1	1	7	4	-	-	-
Figure 2d	0	0	1	10	1	0.06	2.04	0.99
	0	0.25	1	10	1.75	0.19	2.22	0.99
	0	0.5	1	10	2.5	-	-	-
	0	1	1	10	4	-	-	-
Figure 3a	0.25	0	1	5	1.25	-	-	-
	0.25	0	1	7	1.25	0.33	2.25	0.98
	0.25	0	1	8.5	1.25	0.27	14.3	0.98
	0.25	0	1	10	1.25	0.10	0.82	0.93
Figure 3b	0	0.25	1	5	1.75	-	-	-
	0	0.25	1	7	1.75	-	-	-
	0	0.25	1	8.5	1.75	-	-	-
	0	0.25	1	10	1.75	0.19	2.22	0.99
Figure 4	0.3	0	0.7	7	1	0.16	2.38	0.99
	0	0.3	0.1	7	1	0.03	0.12	0.92
	0	0	1	7	1	0.10	2.74	0.98

k_1, the first-order retention coefficient; S_{max}/C_o normalized maximum solid-phase concentration of deposited NPs; R^2, Pearson's correlation coefficient.
